# Key features of *mcr-1*-bearing plasmids from *Escherichia coli* isolated from humans and food

**DOI:** 10.1186/s13756-017-0250-8

**Published:** 2017-09-06

**Authors:** Katrin Zurfluh, Magdalena Nüesch-Inderbinen, Jochen Klumpp, Laurent Poirel, Patrice Nordmann, Roger Stephan

**Affiliations:** 1 Institute for Food Safety and Hygiene, Vetsuisse Faculty University of Zurich, Winterthurerstrasse 272, 8057 Zurich, Switzerland; 2 Institute of Food, Nutrition and Health, ETH Zurich, Schmelzbergstr. 7, 8092 Zurich, Switzerland; 3 Emerging Antibiotic Resistance, Medical and Molecular Microbiology Unit, Department of Medicine, University of Fribourg, Fribourg, Switzerland; 4 National Reference Center for Emerging Antibiotic Resistance, University of Fribourg, Fribourg, Switzerland

**Keywords:** Colistin, *Mcr-1*, Plasmid, IS*Apl1*

## Abstract

**Background:**

*Mcr-1*-harboring Enterobacteriaceae are reported worldwide since their first discovery in 2015. However, a limited number of studies are available that compared full-length plasmid sequences of human and animal origins.

**Methods:**

In this study, *mcr-1*-bearing plasmids from seven *Escherichia coli* isolates recovered from patients (n = 3), poultry meat (n = 2) and turkey meat (n = 2) in Switzerland were further analyzed and compared. Isolates were characterized by multilocus sequence typing (MLST). The *mcr-1*-bearing plasmids were transferred by transformation into reference strain *E. coli* DH5α and MCR-1-producing transformants were selected on LB-agar supplemented with 2 mg/L colistin. Purified plasmids were then sequenced and compared.

**Results:**

MLST revealed six distinct STs, illustrating the high clonal diversity among *mcr-1-*positive *E. coli* isolates of different origins. Two different *mcr-1*-positive plasmids were identified from a single *E. coli* ST48 human isolate. All other isolates possessed a single *mcr-1* harboring plasmid. Transferable IncI2 (size ca. 60–61 kb) and IncX4 (size ca. 33–35 kb) type plasmids each bearing *mcr-1* were found associated with human and food isolates. None of the *mcr-1*-positive IncI2 and IncX4 plasmids possessed any additional resistance determinants. Surprisingly, all but one of the sequenced *mcr-1*-positive plasmids lacked the IS*Apl1* element, which is a key element mediating acquisition of *mcr-1* into various plasmid backbones.

**Conclusions:**

There is strong evidence that the food chain may be an important transmission route for *mcr-1*-bearing plasmids. Our data suggest that some “epidemic” plasmids rather than specific *E. coli* clones might be responsible for the spread of the *mcr-1* gene along the food chain.

## Background

The increasing number of multidrug-resistant Gram-negative bacteria and the lack of novel antimicrobials has led to the reintroduction of polymyxins as last-resort antimicrobials in human medicine, although once avoided because of its nephro- and neurotoxicity [1, 2]. By contrast, in veterinary medicine, colistin is still widely used for the treatment of diarrhea in food-producing animals such as calves and pigs in most countries [3]. Until late 2015, only chromosomally-encoded mechanisms of resistance to polymyxins were known [4]. The mobile colistin resistance gene, *mcr-1,* was first described on a conjugative IncI2 plasmid from Chinese isolates. It encodes a phosphoethanolamine transferase that adds phosphoethanolamine to the lipid A [5]. Retrospective studies performed worldwide revealed that the gene had been circulating undetected for at least twenty years and animals have been suggested to be its main reservoir [6]. The dissemination of *mcr-1* is associated with a large variety of plasmids including incompatibility groups IncI2, IncX4, IncF, IncHI1, IncHI2, IncP and IncY [7–10]. Most of these groups are well known to be involved in the spread of a diversity of antibiotic resistance genes in Enterobacteriaceae.

The aim of this study was to characterize *mcr-1-*bearing plasmids from *E. coli* originating from humans and food isolated at the same location (Switzerland) in order to improve the understanding of the epidemiology and spreading potential of the *mcr-1* gene.

## Methods

In total, seven *E. coli* isolates harboring *mcr-1* plasmids were used in the present study, including one uropathogenic *E. coli* (UPEC) isolate recovered from human urinary tract infection (CDF8), two isolates from humans with diarrhea and history of travel to Asia (ColR598 and ColR644SK1), and two isolates respectively from retail poultry meat (PC11 and PF11) and retail turkey meat (PF52 and PF91). UPEC strain CDF8 was obtained from a patient hospitalized in Switzerland in 2016 (unpublished) and food isolates PC11, PF11, PF52 and PF91 had been isolated in 2016 from food imported from Germany and sold in retail stores in Switzerland [11] ColR598 and ColR644SK1 were obtained from a stool sample screening from patients with diarrhea during the June to December 2016 period. Briefly, a total of 320 non-duplicate samples were screened for the presence of colistin-resistant Enterobacteriaceae by enriching one loopful of stool in 5 ml *Enterobacteriaceae* enrichment (EE) broth (BD, Franklin Lakes, NJ, USA) for 24 h at 37 °C, followed by streaking one loopful onto LB agar plates containing 4 mg/L colistin, 10 mg/L vancomycin and 5 mg/L amphotericin B for selection of colistin-resistant Gram-negative bacteria. The isolates were identified using API ID 32 E (bioMérieux, Marcy l’Etoile, France) and analyzed for the presence of *mcr-1* by PCR as described previously [5]. Minimal inhibitory concentration of colistin was determined for *mcr-1*-positive isolates using broth dilution tests as recommended by EUCAST. Moreover, isolates were subjected to susceptibility testing against 13 antimicrobial agents by the disc diffusion method according to CLSI protocols and evaluated according to CLSI criteria [12].

Multilocus sequence typing (MLST) was performed as described previously [13], and isolates were assigned to sequence types (ST) and clonal complexes (CC) according to the Achtman scheme (http://mlst.ucc.ie/mlst/dbs/Ecoli).

The *mcr-1*-positive plasmids were extracted using the Qiagen Midi kit (Qiagen, Hombrechtikon, Switzerland) and transferred by transformation using electroporation into *E. coli* DH5α. Colistin-resistant transformants were selected on LB-agar supplemented with 2 mg/L colistin (Sigma-Aldrich, Buchs SG, Switzerland). The *mcr-1* plasmids were extracted using the Large-Construct Kit (Qiagen, Hombrechtikon, Switzerland) according the manufacturer’s protocol and sequenced on a PacBio RS2 device (Pacific Biosciences, Menlo Park, USA) with a 10 kb size-selected insert library and P6/C4 chemistry. De novo assembly (using the HGAP3 algorithm) was performed using SMRTanalysis version 2.3.0 (Pacific Biosciences). The HGAP3 settings were kept at the defaults, except for the expected genome size, which was set between 50 kb and 100 kb. The plasmid sequence was automatically annotated using the online Rapid Annotation Subsequencing Technology (RAST) [14] and CLC Main Workbench Version 7.8.1 (CLC bio, Aarhus, Denmark). Automated annotation was manually refined using the BLASTn and BLASTp programs (http://blast.ncbi.nlm.nih.gov/Blast.cgi).

## Results and discussion

The results of this study analyzing seven distinct *mcr-1*-harbouring isolates of different sources are summarized in Table 1. Noticeably, all *mcr-1*-harbouring isolates were *E. coli* that correspond to the most important reservoir of MCR-1 producers identified so far. MLST analysis did not show any close clonal relationship between the seven *mcr-1-*positive *E. coli* isolates, suggesting that the dissemination of the *mcr-1* gene is so far not primarily associated with any specific clonal lineage.

**Table 1:** Features of the eight *mcr-1*-harboring plasmids from seven *E. coli* isolated from humans and from food

Host strain ID	Origin	Source	ST (CC)	MIC colistin [mg/L]	Resistance profile	*mcr-1* harbouring plasmid	Plasmid size (bp)	Inc group	*Mcr-1* gene cassette	Additional resistance genes on the *mcr-1* harbouring plasmid	Reference
PC11	Chicken	Meat	ST1251	8	AM, CF	pPC11	59.830	IncI2	IS*Apl*1-*mcr-1*-orf	none	[11]
PF11	Chicken	Meat	ST156 (CC156)	8	AM, CF, NA, CIP, TE	pPF11	33.308	IncX4	*mcr-1*-orf	none	[11]
PF52	Turkey	Meat	ST58 (CC155)	4	AM, CF, SMZ	pPF52	33.300	IncX4	*mcr-1*-orf	none	[11]
PF91	Turkey	Meat	ST1431	8	AM, CF, TE, C, SMZ	pPF91	33.310	IncX4	*mcr-1-*orf	none	[11]
CDF8	Human	UTI	nd	4	AM, CZ, CTX, FEP, NA, CIP, S, K	pCDF8	33.660	IncX4	*mcr-1*-orf	none	this study
ColR598	Human	Diarrhea	ST48 (CC10)	4	NA, CIP, TE	pColR598_1	33.299	IncX4	*mcr-1*-orf	none	this study
ColR598	Human	Diarrhea	ST48 (CC10)	4	NA, CIP, TE	pColR598_2	60.939	IncI2	*mcr-1*-orf	none	this study
ColR644SK1	Human	Diarrhea	ST117	4	AM, CF, TE, C, SMZ, TMP	pColR644SK1	60.952	IncI2	*mcr-1*-orf	none	this study

*Abbreviations*: *CC* clonal complex; *Inc.* plasmid incompatibility group; *ST* sequence type; *UTI* urinary tract infection, not determined; *MIC* minimal inhibitory concentration; *AM* ampicillin; CF cephalothin; *CZ* cefazolin; *CIP* ciprofloxacin; *NA* nalidixic acid; *K* kanamycin; *S* streptomycin; *SMZ* sulfamethoxazole; *TMP* trimethoprim; *TE* tetracycline; *C* chloramphenicol

**Fig. 1:**
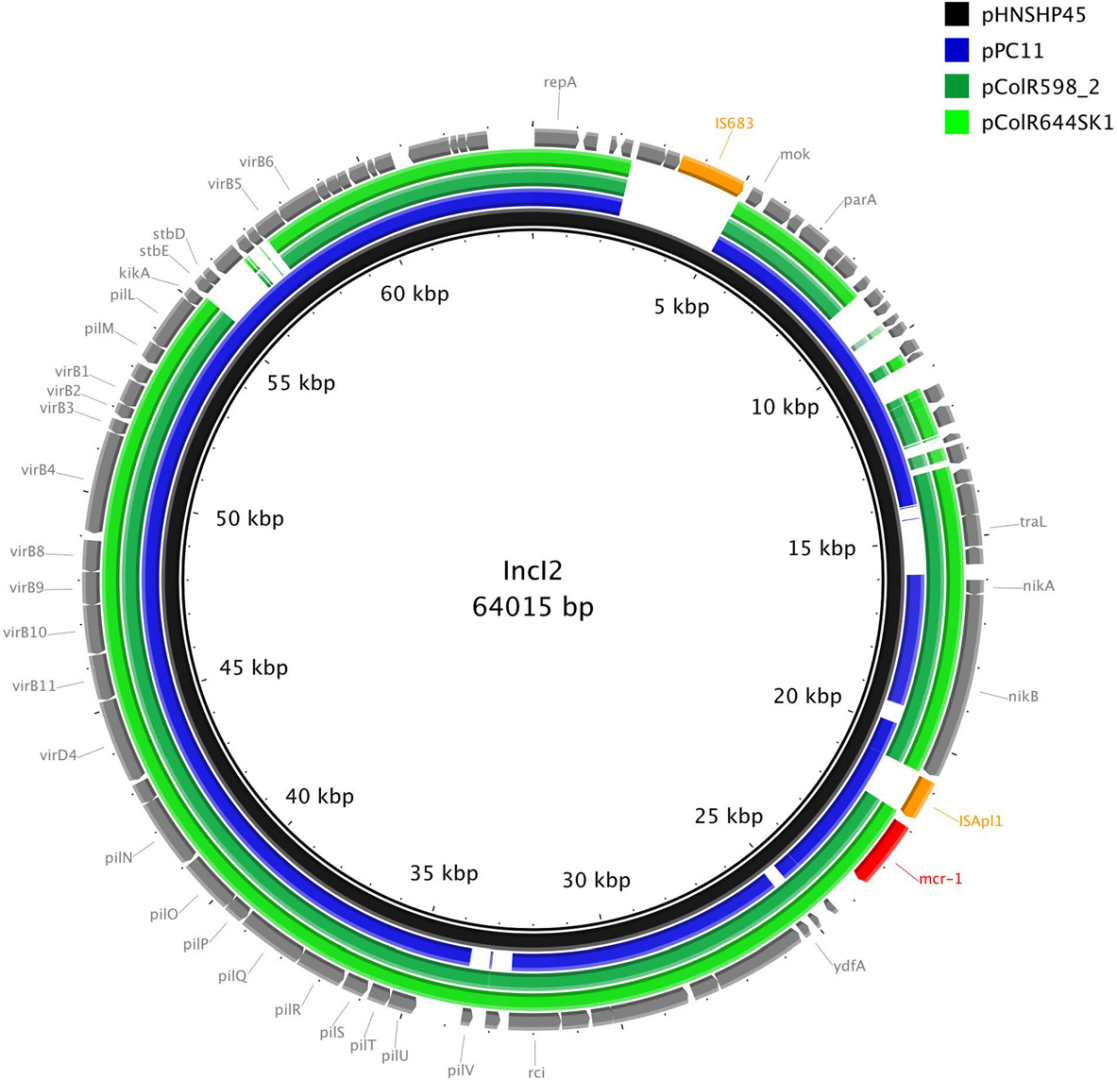
Sequencing alignment of IncI2-type *mcr-1*-harboring plasmids. The first *mcr-1*-harboring plasmid, pHNSHP45 (Accession-Nr. KP347127), which was isolated in China, was used as reference plasmid (black circle). The outmost circle in grey arrows shows the annotations of the reference plasmid. The insertions element and the *mcr-1* gene were highlighted in orange and red arrows, respectively. Gaps indicate regions that were missing in the respective plasmid compared to the reference plasmid

From the seven *E. coli* isolates, a total of eight *mcr-1*-bearing plasmids was recovered, with the human isolate ColR598 yielding two distinct *mcr-1*-positive plasmids. Li and colleagues showed in a recent study that coexistence of two *mcr-1* bearing plasmids seems to be common [15]. Nevertheless, the MIC’s for colistin were not affected by the number of *mcr-1* bearing plasmids present in one isolate [15]. The eight plasmids belonged to two plasmid types that have been often shown to be involved in the spread of *mcr-1* [16]. Three IncI2 plasmids (pPC11, pCoR598_2 and pColR644SK1) were ca. 60 kb in-size, and were similar to pHNSHP45 (Fig. 1), the original sequenced *mcr-1* plasmid published in 2015 [5]. The three IncI2 plasmids shared a common plasmid backbone, however, in the case of pPC11 the *mcr-1* cassette [17] was located in an inverted orientation compared to the others (data not shown). Additionally, the IncI2 plasmids from human isolates (pColR598_2 and pColR644SK1) varied greatly compared to the plasmid pPC11 (poultry isolate) in the shufflon region, which is a clustered inversion region encoding components of the pilV protein involved in plasmid transmission [18]. The components are rearranged by Rci, a recombinase encoded by the *rci* gene (Fig. 1). This observation is in accordance with recently sequenced IncI2 plasmids carrying *mcr-1* detected in *E. coli* from swine and cattle in Japan [19].

**Fig. 2:**
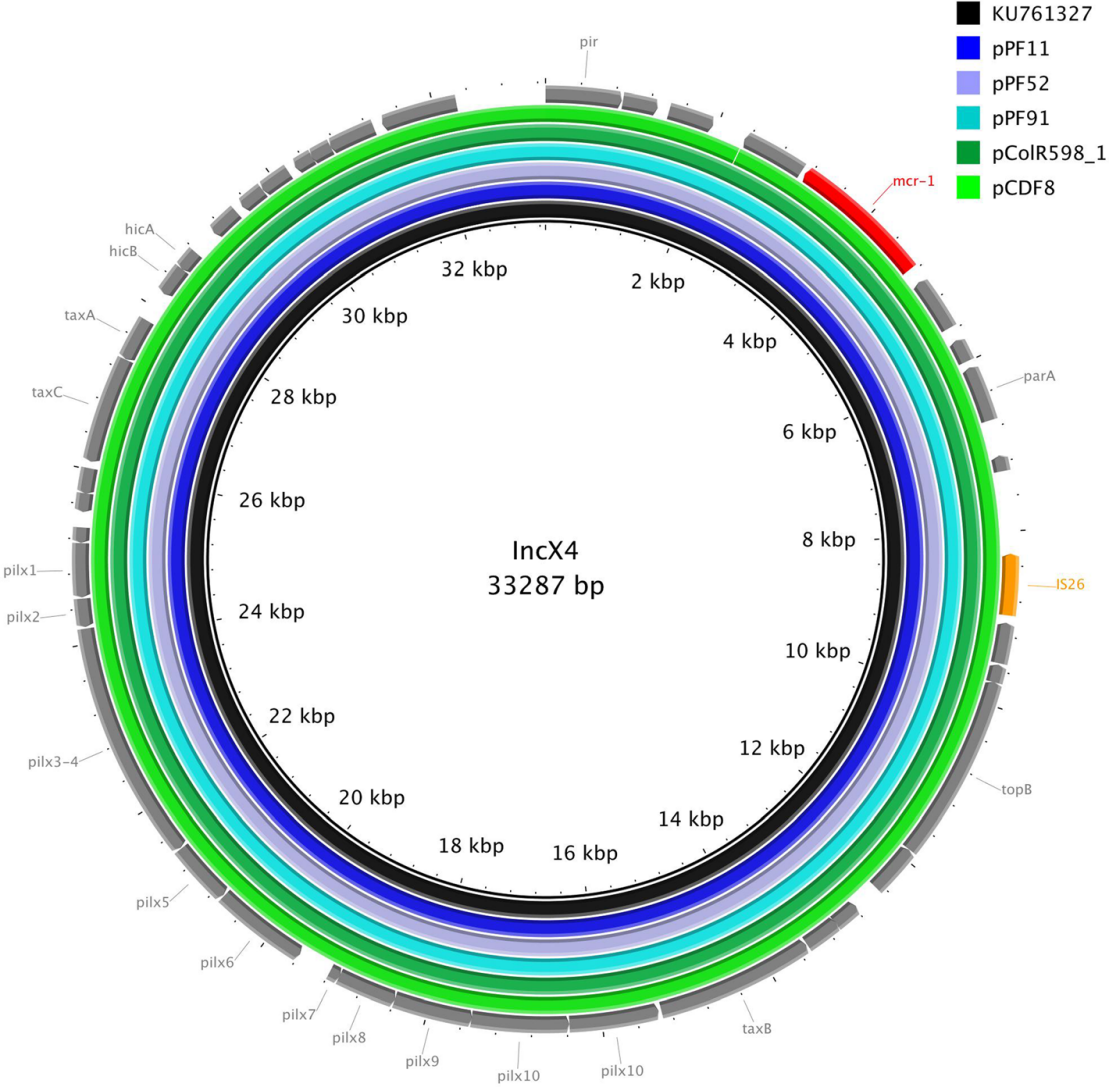
Sequencing alignment of IncX4-type *mcr-1*-harboring plasmids. The *mcr-1* harboring plasmid, pmcr-1_X4 (Accession-Nr. KU761327), which was obtained from two *Klebisiella pneumonia* isolates and one *Escherichia coli* isolate from patients in eastern China and which was one of the first sequenced IncX4 *mcr-1* positive plasmids, was used as reference plasmid (black circle). The outmost circle in grey arrows shows the annotations of the reference plasmid. The insertions element and the *mcr-1* gene were highlighted in orange and red arrows, respectively. The figure indicates the high degree of homology of the *mcr-1* harboring IncX4 plasmids independently of their isolation source and geographical origin

The other five plasmids (pPF11, pPF52, pPF91, pCDF8 and pColR598_1) all belonged to the plasmid incompatibility group IncX4 and were ca. 33 kb in-size. Their sequences varied only by very few nucleotides (≥99% homology), mostly located in non-coding regions (Fig. 2). In the case of pDCF8 the *mcr-1* cassette was located in an inverted orientation compared to the others (data not shown). Of note, those almost identical IncX4 plasmids originate from humans, poultry and turkey meat, illustrating their wide dissemination throughout multiple sources, and providing further evidence of the likely association of *mcr-1*-mediated colistin resistance through food-producing animals.

An open reading frame (*orf*) encoding an hypothetical protein with similarities to a PAP2 superfamily protein was detected immediately downstream of the *mcr-1* gene (both together hereafter referred to as *mcr-1* cassette) was identified on all eight plasmids.

The insertion sequence IS*Apl1*, has been shown to play a key role in the mobilization of *mcr-1* [20], but was absent upstream of *mcr-1* in most of our isolates. Further evidence for the importance of IS*Apl1* in the mobilization of *mcr-1* was the presence of IS*Apl1* next to the *mcr-1* gene on the chromosome of an *E. coli* veal calf isolate from Netherland [21]. Moreover, transposition of the *mcr-1* gene by an IS*Apl1-*made composite transposon was recently demonstrated [17]. Highly similar inverted repeat (IRR) and direct repeat (DR) sequences were identified on IncX4, IncHI1 and IncHI2 backbones immediately downstream of the *mcr-1* cassette, resembling the target insertion site resulting from the IS*Apl1*-mediated transposition [16], although the IS element itself was not always present*.* In a recent study, no putative inverted repeat sequences were identified at the extremities of the *mcr-1* cassette [22]. Furthermore, Snesrud and colleagues [20] proposed the loss of one or both IS*Apl1* elements as an explanation for the minor variations (mismatches and deletions) at the 3’end of the *mcr-1* element. Accordingly, in this study, IS*Apl1* was present only on a single IncI2 plasmid (pPC11) and was located upstream of the *mcr-1* gene.

Noticeably, none of the sequenced plasmids carried additional antibiotic resistance determinants. This is in agreement with other observations [5, 9, 22] and appears to be quite a specificity to the *mcr-1* gene, considering that most antibiotic resistance plasmids often carry multiple resistance genes. It is therefore tempting to speculate that this specificity is related to selection of those MCR-1 determinants by treatment containing polymyxins in animals. Moreover, the food samples described in this study originated from Germany, a country with high use of colistin in animal husbandry, and both humans with diarrhea had visited countries in Asia, where colistin is applied widely to treat animals [3]. However, the extended-spectrum ß-lactamase gene *bla*_CTX-M-64_ has recently been detected on an *mcr-1*-harboring IncI2 plasmid [23]. There are some further data were the *mcr-*1 gene was located on large multidrug resistance plasmids for example in combination with extended-spectrum beta-lactamase genes [9, 15, 24]. In these studies *mcr-1* was mainly harboured on IncHI2 or IncF plasmids. It is to be expected that *mcr-1* harboring plasmids co-harboring resistances to antimicrobials crucial to human treatment become more frequent in future.

## Conclusion

Transferable IncI2 and IncX4 type plasmids harbouring *mcr-1* were found in *E. coli* of different clonal backgrounds isolated from humans and from food. The high similarity between the plasmids belonging to the same incompatibility groups shows that these “epidemic” plasmids may be responsible for the spread of the *mcr-1* gene along the food chain and humans, rather than single specific *E. coli* clones. A single strain may even contain more than one *mcr-1*-harboring plasmid. Further studies are needed in order to determine the mechanisms that lead to the acquisition or even accumulation of *mcr-1*-harboring plasmids within Enterobacteriaceae.

## Abbreviations

*bla*: ß-lactamase gene
CC: Clonal complex
EE: *Enterobacteriaceae* enrichment
Inc: Plasmid incompatibility group
MLST: Multilocus sequence type
NENT: National Centre for Enteropathogenic Bacteria and *Listeria*
ST: Sequence type
UPEC: Uropathogenic *E. coli*
UTI: Urinary tract infection
